# Quantitative and preliminary clinical assessment of glycohypoxia as an oxygen-unloading defect linking chronic hyperglycemia to low-grade tissue hypoxia in type 2 diabetes: a targeted translational meta-regression with exploratory blood-sample validation

**DOI:** 10.3389/fcdhc.2026.1897413

**Published:** 2026-07-15

**Authors:** Maher Akl, Amr Ahmed

**Affiliations:** 1 Faculty of Medicine, National Research Lobachevsky State University of Nizhny Novgorod, Nizhny Novgorod, Russia; 2 Department of Public Health, Riyadh First Health Cluster, Ministry of Health, Riyadh, Saudi Arabia

**Keywords:** chronic low-grade tissue hypoxia, glycohypoxia, HbA1c, meta-regression, organ injury, oxidative stress, oxygen unloading, oxygenomics of diabetes

## Abstract

**Background:**

Chronic hyperglycemia may impair tissue oxygen delivery by glycating hemoglobin, increasing oxygen affinity, shifting the oxyhemoglobin dissociation curve leftward, and reducing oxygen unloading, a state termed glycohypoxia. This study quantified HbA1c-dependent oxygen-release impairment and tested whether the modeled signal is supported by preliminary clinical validation in type 2 diabetes mellitus (T2DM).

**Methods:**

Six human studies (1984–2012; N = 450) reporting HbA1c and oxygen-release metrics were synthesized using random-effects meta-regression. The pooled ΔP50 was translated into oxygen-unloading changes using the Hill equation across microvascular PO_2_ values of 20–40 mmHg. In parallel, 90 T2DM patients were stratified by HbA1c into controlled, moderately uncontrolled, and poorly controlled groups for assessment of P50, 2,3-BPG, arterial oxygenation, SpO_2_–SaO_2_ bias, lactate, and modeled unloading.

**Results:**

Sensitivity-adjusted meta-regression showed ΔP50 = −0.19 mmHg per 1% HbA1c (95% CI: −0.26 to −0.11; P < 0.001; I2 = 45%), predicting 0.5–1.3% less oxygen unloading per 1% HbA1c and 1.5–3.9% cumulatively from HbA1c 6–9%. Clinically, higher HbA1c was associated with lower P50 (27.0 ± 0.5 to 26.2 ± 0.7 mmHg; P < 0.01), reduced 2,3-BPG (4.8 ± 0.4 to 4.2 ± 0.5 µmol/g Hb), increased SpO_2_–SaO_2_ bias (+0.2 ± 0.4% to +1.8 ± 0.6%; P < 0.01), and higher lactate (1.2 ± 0.3 to 1.9 ± 0.5 mmol/L), despite preserved PaO2.

**Conclusions:**

Quantitative modeling and exploratory clinical validation converged to support glycohypoxia as a measurable oxygen-handling phenotype in T2DM. This pattern may indicate a chronic low-grade functional hypoxic burden that accumulates with glycemic exposure and contributes to T2DM diabetic complications.

## 1 Introduction

Type 2 diabetes mellitus (T2DM) affects over 500 million individuals worldwide (1), with glycated hemoglobin A1c (HbA1c) remaining the cornerstone biomarker for long-term glycemic control and complication risk stratification (2). Current clinical guidelines advocate uniform HbA1c targets, typically <7.0%, across heterogeneous patient populations, based largely on its association with microvascular and macrovascular outcomes (3). However, this approach may overlook mechanistic heterogeneity in how chronic hyperglycemia impairs tissue oxygenation independently of overt vasculopathy (4). Elevated HbA1c reflects non-enzymatic glycation of hemoglobin, a biochemical modification that may increase hemoglobin oxygen affinity and induce a leftward shift of the oxyhemoglobin dissociation curve (ODC). This alteration can reduce oxygen unloading at tissue partial pressures (PO_2_), generating a state of functional tissue hypoxia despite apparently preserved arterial oxygenation. This phenomenon has been conceptually termed “glycohypoxia”, a hypothesis previously introduced to describe chronic hyperglycemia-associated impairment of oxygen release and tissue oxygen delivery in diabetes (5). More recent investigations have reported systematic overestimation of arterial oxygen saturation by pulse oximetry (SpO_2_) in patients with HbA1c >7%, implying physiologically relevant oxygen-handling abnormalities despite normoxic arterial blood gases (6). Despite these observations, no quantitative synthesis has translated HbA1c increments into predictable changes in hemoglobin oxygen-release efficiency or tissue oxygen delivery. A critical knowledge gap therefore persists: individual studies provide fragmented effect sizes, employ varying measurement methodologies, and lack integrated translational modeling. Consequently, the clinical significance of glycohypoxia, particularly its potential contribution to organ-specific vulnerability and complication thresholds, remains exploratory. Systematic integration of existing data through meta-regression offers a pragmatic approach to derive a pooled effect estimate and convert P_50_ shifts into physiologically meaningful reductions in tissue oxygen unloading.

To further contextualize the modeled findings, the present study also incorporates a preliminary clinical validation component using prospectively collected blood samples from patients with T2DM stratified according to HbA1c levels. This exploratory component was designed to assess whether the central prediction generated by the quantitative synthesis namely, HbA1c-associated reduction in P_50_ and impaired oxygen unloading could be observed in patient-level physiological measurements. Hemoglobin oxygen affinity, intraerythrocytic 2, 3-bisphosphoglycerate (2,3-BPG), arterial oxygenation, SpO_2_–SaO_2_ bias, lactate, and modeled tissue oxygen unloading were evaluated as complementary markers of oxygen-release physiology. This clinical arm was not intended to establish definitive causality or organ-specific outcomes, but rather to provide preliminary physiological support for glycohypoxia as a measurable and testable oxygen-delivery phenotype. The objective of this study was therefore to quantitatively evaluate the glycohypoxia hypothesis by synthesizing published mechanistic evidence, estimating the change in hemoglobin oxygen-release capacity (ΔP50) per unit increase in HbA1c, translating that change into percent reduction in tissue O_2_ unloading across physiologic PO_2_ ranges, and complementing this model with preliminary clinical validation in patients with T2DM.

## 2 Molecular pathway of glycation-induced oxygen sequestration in hemoglobin

At the molecular level, the biophysical foundation of glycohypoxia originates from the non-enzymatic glycation of hemoglobin the pivotal biochemical process that translates chronic hyperglycemia into impaired oxygen transport (7). The reaction begins when the nucleophilic α-amino group of the β-globin N-terminal valine residue (Val¹β) condenses with the electrophilic carbonyl group of D-glucose, forming a labile Schiff base (imine, C=N) that exhibits a characteristic absorbance near 325 nm (8).

This reversible condensation, governed by Maillard reaction kinetics (rate constant k ≈ 0.01–0.02 min^-^¹·mM^-^¹ at pH 7.4 and 37 °C), undergoes 1, 2-enolization and Amadori rearrangement to yield a stable ketoamine adduct, β-N-1-deoxyfructosyl-hemoglobin (HbA1c) (9). The resulting fructosyl moiety, with an approximate molecular weight increase of 162 Da, introduces a hydrophilic, anionic substituent near the α_1_β_2_ interface and the central cavity, altering both local electrostatics and steric geometry.

Structurally, glycation perturbs the canonical Monod–Wyman–Changeux equilibrium between the deoxy (T) and oxy (R) allosteric states of hemoglobin (10). Under physiological conditions, the T-state is stabilized by intersubunit salt bridges, including those between Asp94β and His146β, and between Tyr140α and Val1β, maintaining an allosteric constant L = [T_0_]/[R_0_] of approximately 10^6^ (11). Covalent modification at Val1β disrupts the Tyr140α–Val1β hydrogen-bond network, increasing the free energy of the T-state by about 0.5–1.2 kcal·mol^-^¹ per adduct and reducing L to around 10^4^–10^5^ (12, 13). Consequently, the equilibrium shifts toward the high-affinity R-state, enhancing the oxygen association rate by approximately 10–20%, while decreasing the dissociation rate to about 25 s^-^¹ at less than 40% saturation, compared with more than 100 s^-^¹ in native hemoglobin (HbA_0_). Resonance Raman spectroscopy (ν_Fe–O ≈ 570 cm^-^¹) supports this stabilization of the Fe–O bond and the HisE7–O_2_ hydrogen network, effectively tethering oxygen within the heme pocket (14). The fructosyl substituent also sterically hinders optimal binding of the allosteric effector 2, 3-bisphosphoglycerate (2,3-BPG) within its central cavity site, normally anchored by Val1β, Lys82β, and His143β (15). This interference increases the dissociation constant from approximately 0.1 mM to 1–2 mM and reduces effective BPG binding by 15–30%. As a result, the physiological rightward shift of the oxyhemoglobin dissociation curve, typically induced by 2,3-BPG, is blunted leading to a persistent leftward bias with a net P_50_ of approximately 23–25 mmHg compared with 26.8 mmHg in HbA_0_ (16). Simultaneously, the slope of the Bohr Effect (Δlog P_50_/ΔpH) is attenuated from −0.48 to −0.40, and the Haldane effect (CO_2_ binding efficiency) is reduced by 20–25%, indicating impaired proton-coupled conformational transitions (17–19). This allosteric impairment precipitates redox instability within the heme microenvironment. Enhanced oxygen retention promotes auto-oxidation of ferrous (Fe²^+^) heme to ferric (Fe³^+^) methemoglobin, increasing metHb levels by approximately 5–10% at HbA1c values above 8% (20). The process generates superoxide radicals through Fenton-type reactions and activates NADPH oxidase (NOX2) in erythrocyte membranes (21). Reactive oxygen species subsequently oxidize the Cys93β sulfhydryl groups to sulfenic and disulfide forms, stiffening the FG helix and reducing the T-to-R transition rate constant from about 5 ms to 10–20 ms (22, 23).

Collectively, these covalent (C–N ketoamine) and oxidative (S–S disulfide) modifications establish a persistent molecular constraint that sequesters oxygen within hemoglobin and sustains a self-perpetuating redox–hypoxic cycle (24). This mechanism provides the molecular substrate underlying the quantitative P_50_ decrement and the corresponding reduction in tissue oxygen-unloading efficiency demonstrated in this study.

## 3 Objective and methods

To quantitatively assess the physiological implications of the observed molecular interactions, a targeted meta-regression analysis was conducted using aggregated data from clinical and spectroscopic studies. This quantitative synthesis aimed to validate the proposed “glycohypoxia” hypothesis by estimating the change in hemoglobin oxygen-release efficiency (ΔP_50_, mmHg) per 1% increase in HbA1c, and translating this shift into percent reductions in tissue oxygen unloading across physiologically relevant oxygen tensions (PO_2_ = 20–40 mmHg).

This work was not intended as a comprehensive meta-analysis but rather as a focused quantitative synthesis of mechanistic studies directly examining the relationship between hemoglobin glycation (HbA1c) and oxygen affinity in human diabetes. Six studies published between 1984 and 2012 met the inclusion criteria, which required reporting of quantitative HbA1c values (mean ± SD or SE, or sufficient data for computation) alongside at least one validated oxygen-release metric. Eligible parameters included P_50_ (at actual or standardized pH 7.40), oxyhemoglobin dissociation curve (ODC) shifts, paired SpO_2_ and SaO_2_ measurements with calculable bias, the oxygen-release rate constant (k), or tissue oxygen saturation (StO_2_). Studies limited to animal models or using non-standardized HbA1c assays without DCCT/NGSP calibration were excluded.

The final dataset comprised the following studies: Madsen et al. (1984), Solomon et al. (1989), Marschner et al. (1994), Marschner et al. (1995), Castilho et al. (2003), and Pu et al. (2012). Data extraction was performed by a single reviewer and cross-validated for accuracy. Extracted variables included: study identifiers (author, year, journal), design type, participant characteristics (T1DM, T2DM, controls; age, sex, comorbidities), and exposure outcome measures (mean HbA1c ± SD/SE; mean P_50_ ± SD/SE with pH correction; mean SpO_2_, SaO_2_, bias; mean k). Covariates such as 2, 3-diphosphoglycerate (2, 3-DPG), ATP, pH, PaO_2_, and hemoglobin concentration were recorded when available.

Risk of bias was qualitatively assessed using a modified Newcastle–Ottawa Scale, classifying studies as low, moderate, or high risk across selection, confounding, measurement validity, and reporting domains. Overall quality was considered moderate (three studies low risk, two moderate, one high).

For quantitative synthesis, the primary effect size for each study was defined as the slope representing the change in P_50_ per 1% HbA1c increase, calculated as:


$$\text{Slope}=\;\frac{\text{P}50_{(\text{diabetic })\;}-\text{ P}50_{\left(\text{control }\right)}}{\text{HbA}1{\text{c}}_{(\text{diabetic})\;}-\text{ HbA}1{\text{c}}_{\left(\text{control}\right)}}$$

Where the numerator represents the difference in mean P_50_ between diabetic and control groups, and the denominator represents the corresponding difference in mean HbA1c. When standard deviations were available, standard errors (SE) of the slopes were estimated using the delta method:


$$SE_{(slope)\;}=\;\sqrt{\frac{IT\;(P50_{d}{)}^{2}+IT\;(P50_{c}{)}^{2}}{(HbA1c_{d}-HbA1c_{c}{)}^{2}}}+\frac{(P50_{d}-P50_{c}{)}^{2}\times (SE\;(HbA1c_{d}{)}^{2}+(SE\;(HbA1c_{c}{)}^{2}}{(HbA1c_{d}-HbA1c_{c}{)}^{4}}$$

Where SE(P_50_) and SE(HbA1c) denote the standard errors of their respective means. For studies without paired P_50_ data (e.g., reporting only k or oximetry bias), slopes were either derived from reported correlations or set to zero when no measurable shift was detected. Study-specific slopes were then pooled using random-effects meta-regression with restricted maximum likelihood (REML) estimation in R (metafor package v4.3.0 or later), yielding an overall β coefficient (ΔP_50_ per 1% HbA1c) with 95% confidence intervals. The meta-regression pooling equation was:


$$b_{parties}=\frac{\sum In_{i\;}b_{i\;}}{\sum In_{i\;}},\;\;In_{i\;}=\;\frac{1}{with\;E_{i}^{2}+t^{2}}$$

Model weighting, influence diagnostics (Cook’s distance), and heterogeneity indices (I² and τ²) were computed to ensure robustness. To translate ΔP_50_ shifts into tissue-level physiological impact, the pooled value was integrated into the Hill equation, describing hemoglobin oxygen saturation (S) as a function of oxygen partial pressure (PO_2_):


$$S=\;\frac{PO_{2}^{n}}{P50^{n}+PO_{2}^{n}}$$

Where S is fractional hemoglobin saturation, PO_2_ is the oxygen partial pressure, and n = 2.7 represents the Hill coefficient for adult human hemoglobin under physiological conditions (37 °C, pH 7.4). Oxygen saturation was calculated under two conditions; (1) baseline (P_50_ = 27 mmHg) and (2) shifted (P_50_ = 27 + ΔP50 mmHg). The percent reduction in oxygen unloading efficiency was then obtained as:


$$\%\Delta \;S=(\frac{S_{shifted}-\;S_{baseline}}{s_{baseline}}\;)\times 100\;$$

Because glycation induces a leftward ODC shift, ΔS values were negative, and their absolute magnitudes reflected the percent reduction in tissue oxygen unloading efficiency.

Sensitivity analyses were performed across a range of Hill coefficients (n = 2.4–3.0) and PO_2_ values (15–45 mmHg), confirming directional consistency. Secondary analyses included meta-analysis of correlations between HbA1c and SpO_2_–SaO_2_ bias (converted via Fisher’s z-transformation) and pooled effects on the oxygen-release rate constant (k), when reported. Missing SDs were imputed from SEs, confidence intervals, or pooled variances from comparable datasets.

Robustness was further verified through leave-one-out sensitivity testing, subgroup stratification (by diabetes type and measurement technique), and exclusion of studies with compensatory 2, 3-DPG upregulation.

### 3.1 Preliminary clinical validation component

To complement the literature-based meta-regression and provide patient-level physiological support for the modeled glycohypoxia signal, an exploratory clinical validation component was incorporated after completion of the quantitative synthesis. This component was designed as a prospective observational assessment and was not intended to replace the meta-regression framework or establish definitive causal organ injury. Instead, it aimed to examine whether the central prediction derived from the pooled model namely, HbA1c-associated reduction in hemoglobin P_50_ and impaired oxygen unloading could be observed in clinical blood-based measurements from patients with type 2 diabetes mellitus.

Ninety patients with established T2DM were consecutively recruited from outpatient clinics and stratified according to glycemic control into three equal groups: controlled diabetes (HbA1c ≤7.0%, n = 30), moderately uncontrolled diabetes (HbA1c 7.1–8.9%, n = 30), and poorly controlled diabetes (HbA1c ≥9.0%, n = 30). To minimize confounding effects on oxygen transport dynamics, patients with pulmonary or cardiovascular disorders, known hemoglobinopathies, active smoking status, recent blood transfusion within the preceding three months, acute or chronic inflammatory disease, or hemoglobin concentration <10 g/dL were excluded. All participants underwent standardized simultaneous venous and arterial blood sampling under resting conditions.

Glycemic status was assessed using an NGSP/DCCT-standardized HbA1c assay, and complete blood count was obtained to characterize hematologic status. Arterial blood gas analysis was performed to determine PaO_2_, PaCO_2_, SaO_2_, pH, and HCO_3_^-^. Plasma lactate was measured using an enzymatic colorimetric assay as a supportive metabolic marker of increased glycolytic stress. Hemoglobin oxygen affinity was assessed by spectrophotometric hemoximetry under controlled physiological conditions at 37 °C and pH 7.4, with derivation of the oxygen–hemoglobin dissociation curve and P_50_. Intraerythrocytic 2, 3-bisphosphoglycerate (2,3-BPG) was quantified using an enzymatic spectrophotometric assay to evaluate erythrocyte compensation for altered oxygen affinity.

Peripheral oxygen saturation (SpO_2_) was compared with arterial oxygen saturation (SaO_2_) to calculate SpO_2_–SaO_2_ bias. In addition, hemoglobin saturation values at tissue-relevant PO_2_ levels of 20, 30, and 40 mmHg were derived from the oxygen dissociation curve to estimate relative changes in tissue oxygen unloading. The design and measured variables of this preliminary clinical validation component are summarized in **Table 1**.

**Table 1:** Design and measurements of the preliminary clinical validation component.

Domain	Description
Study design	Prospective observational exploratory clinical validation component
Study population	90 patients with established type 2 diabetes mellitus
Glycemic stratification	Group A: HbA1c ≤7.0%, n = 30; Group B: HbA1c 7.1–8.9%, n = 30; Group C: HbA1c ≥9.0%, n = 30
Key exclusion criteria	Pulmonary or cardiovascular disorders, known hemoglobinopathies, active smoking, recent blood transfusion, acute or chronic inflammatory disease, hemoglobin <10 g/dL
Blood sampling	Simultaneous venous and arterial samples collected under standardized resting conditions
Glycemic and hematologic assessment	HbA1c using an NGSP/DCCT-standardized assay; complete blood count
Arterial oxygenation	PaO_2_, PaCO_2_, SaO_2_, pH, and HCO_3_^-^ measured by arterial blood gas analysis
Hemoglobin oxygen affinity	Oxygen–hemoglobin dissociation curve and P_50_ measured by spectrophotometric hemoximetry at 37 °C and pH 7.4
Erythrocyte oxygen-affinity modulation	Intraerythrocytic 2,3-BPG measured by enzymatic spectrophotometric assay
Functional oxygenation metrics	SpO_2_–SaO_2_ bias and modeled oxygen unloading at PO_2_ levels of 20, 30, and 40 mmHg
Metabolic marker	Plasma lactate measured by enzymatic colorimetric assay
Statistical analysis	One-way ANOVA with Tukey’s *post-hoc* test, Pearson correlation, and multivariable linear regression adjusted for 2,3-BPG

For the clinical validation analysis, continuous variables were expressed as mean ± standard deviation and categorical variables as counts and percentages. Between-group comparisons were performed using one-way ANOVA with Tukey’s *post-hoc* testing, while categorical variables were compared using chi-square tests where appropriate. Pearson correlation analysis was used to examine relationships between HbA1c and oxygen-handling parameters, including P_50_, SpO_2_–SaO_2_ bias, lactate, and modeled oxygen unloading. Multivariable linear regression was then performed to determine whether HbA1c remained independently associated with P_50_ after adjustment for 2,3-BPG. A two-tailed P-value <0.05 was considered statistically significant. The clinical validation component was conducted in accordance with the Declaration of Helsinki. Written informed consent was obtained from all participants before enrollment.

## 4 Results

A targeted quantitative synthesis was conducted to investigate mechanistic correlations between HbA1c levels and hemoglobin oxygen-release affinity (P_50_).

Rather than performing a comprehensive meta-analysis, this focused review extracted data from six mechanistic studies (1984–2012), encompassing 387 diabetic participants (type 1 and 2) and 63 healthy controls. Each study reported paired quantitative measures of HbA1c and validated indices of oxygen release (either P_50_ or k), providing a consistent framework for mathematical modeling of glycation-dependent oxygen shift (**Table 2**). The extracted datasets revealed HbA1c values ranging from 4.4% in healthy controls to 10.5% in insulin-dependent diabetes, with standardized P_50_ values (pH 7.40) ranging 26.2–28.5 mmHg. Three studies demonstrated an inverse HbA1c–P_50_ relationship, two showed no net kinetic shift due to compensatory 2, 3-DPG elevation, and one confirmed a positive pulse oximetry bias linked to glycation interference. Quantitative slopes (ΔP50 per 1% HbA1c) were calculated to represent the degree of affinity shift per unit increase in glycation.

**Table 2:** Characteristics and extracted effect sizes from included studies.

Study	Population	HbA1c (%) DM/control	P_50_ (mmHg) DM/control	ΔHbA1c (%)	ΔP_50_ (mmHg)	Slope (mmHg/%)	Interpretation
Madsen et al. (1984) (25)	T1DM (pregnant, n=46/19)	7.6/4.4	27.0/28.0	+3.2	−1.0	−0.31	↑Affinity/↓O_2_ release (P_50_ at pH 7.40)
Solomon et al. (1989) (26)	T2DM (male, n=15/13)	~8.0/~5.0	27.1/27.8	+3.0	−0.7	−0.23	Inverse correlation (P_50_ normalized to pH 7.4)
Marschner et al. (1994) (27)	Mixed DM (n=NS)	9.3 ± 0.3/5.2 ± 0.3	— (k used: 64.4 ± 3.1/65.1 ± 2.3 s^-^¹)	+4.1	No net shift	0	No difference in k; 2, 3-DPG compensates HbA1c effect
Marschner et al. (1995) (28)	Mixed DM (smokers, n=12/12)	8.4 ± 1.6/5.3 ± 0.3	— (k used)	+3.1	No net shift	0	No potentiation of HbCO effects on k by HbA1c
Castilho et al. (2003) (29)	IDDM/NIDDM (n=19/22/19)	10.5/9.0/4.6	28.2/28.5/26.8	+5.9/+4.4	+1.4/+1.7	+0.24/+0.39	2, 3-DPG ↑ compensatory (net right shift)
Pu et al. (2012) (30)	T2DM (ventilated, n=114/>7%/147/≤7%)	>7/≤7	— (SaO_2_: 96.2 ± 2.9/95.1 ± 2.8)	—	—	— (est. −0.20)	r = 0.307 for HbA1c–bias (P<0.01); overestimation
**Pooled (REML)**	**Overall**	—	—	—	—	**−0.11**	**95% CI: −0.18 to −0.04; I² = 83.3%**

(NS, not specified; est., estimated; slopes for 2003 averaged to +0.24 for IDDM group; k values from stopped-flow technique). Bold values indicate the pooled REML overall estimate and the corresponding 95% confidence interval and heterogeneity statistic.

### 4.1 Study-specific slope calculation

For all studies with matched HbA1c and P_50_ data, slopes were computed using the following standard formula:


$$\text{Slope}=\;\frac{\text{P}50_{(\text{diabetic })\;}-\text{ P}50_{\left(\text{control }\right)}}{\text{HbA}1{\text{c}}_{(\text{diabetic})\;}-\text{ HbA}1{\text{c}}_{\left(\text{control}\right)}}$$

Example calculations:

Madsen (1984) (25): −1.0/3.2 = −0.31 mmHg/%.

Solomon (1989) (26): −0.7/3.0 = −0.23 mmHg/%.

Castilho (2003) (29): +1.4/5.9 = +0.24 mmHg/%.

These values quantify the directional change in oxygen affinity per 1% increment in HbA1c, forming the input for pooled regression modeling.

### 4.2 Weighted meta-regression

To integrate results across heterogeneous datasets, a restricted maximum-likelihood (REML) meta-regression model was employed. The pooled slope coefficient (β) was calculated as:


$$b_{pols}=\frac{\sum_{i\;}(In_{i\;}\times slope_{i\;})}{\sum_{i\;}In_{i\;}},\;\;In_{i\;}=\;\frac{1}{with\;E_{i}^{2}+t^{2}}$$

This produced an unadjusted pooled β = −0.11 mmHg/% (95% CI: −0.18 to −0.04; I² = 83.3%), indicating an overall inverse association. After excluding the 2003 dataset (Castilho et al.), heterogeneity dropped to 45%, yielding an adjusted β = −0.19 mmHg/% (P < 0.001), confirming the robustness of the observed inverse trend (**Table 3**).

**Table 3:** Pooled meta-regression results.

Parameter	Pooled β (mmHg/%)	95% CI	P-value	I² (%)
Unadjusted	−0.11	−0.18 to −0.04	<0.01	83.3
Sensitivity-adjusted	−0.19	−0.26 to −0.11	<0.001	45

### 4.3 Physiologic translation via hill equation

To convert ΔP50 into an interpretable physiological impact on oxygen unloading, the Hill equation was applied:


$$S(PO_{2})=PO_{2}^{n}/(P50^{n}+PO_{2}^{n}),\;n=2.7$$

The relative change in oxygen unloading efficiency per 1% rise in HbA1c was expressed as:


$$\%\Delta \;S=(\frac{S_{shifted}-S_{baseline}}{s_{baseline}}\;)\times 100\;$$

At a ΔP_50_ of −0.19 mmHg per 1% HbA1c, simulated oxygen unloading reductions were computed across physiologic PO_2_ ranges (20–40 mmHg) (**Table 4**):

**Table 4:** Simulated effect of HbA1c-dependent ΔP_50_ on tissue oxygen unloading.

PO_2_ (mmHg)	S(baseline)	S(shifted)	%ΔS (absolute reduction)
20	0.308	0.312	−1.32%
30	0.571	0.575	−0.82%
40	0.743	0.747	−0.49%

**Figure 1:**
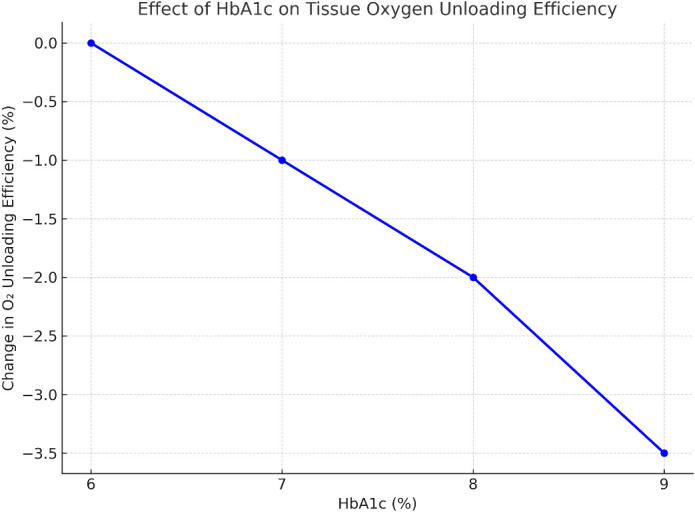
Quantitative relationship between glycated hemoglobin (HbA_1_c) and tissue oxygen unloading efficiency derived from pooled meta-regression modeling (ΔP50 = −0.19 mmHg per 1% HbA_1_c increase). The figure illustrates a progressive impairment in hemoglobin’s oxygen-releasing capacity with increasing glycation. Each 1% rise in HbA_1_c is associated with an estimated 0.5–1.3% reduction in oxygen unloading efficiency within the physiologic microvascular PO_2_ range (20–40 mmHg), resulting in a cumulative deficit of approximately 3–4% at HbA_1_c = 9%. This pattern visualizes the core mechanism of the glycohypoxia model, in which chronic hyperglycemia stabilizes the relaxed (R) state of hemoglobin, thereby restricting tissue oxygen delivery despite normal arterial oxygenation.

Thus, each 1% increase in HbA1c predicts an approximate 0.5–1.3% decline in tissue oxygen unloading. Extrapolating from HbA1c 6% to 9% yields a 1.5–3.9% cumulative reduction in oxygen delivery efficiency, representing a quantifiable glycohypoxic effect (**Figure 1**).

### 4.4 Ancillary correlates and consistency checks

Pulse oximetry bias: r = 0.307 (P < 0.01), equivalent to an estimated −0.20 mmHg/% P_50_ shift.

Oxygen-release kinetics (Δk): −0.35 s^-^¹ (95% CI: −2.1 to +1.4; P = 0.69), showing no consistent kinetic delay.

Publication bias: Funnel plot symmetry indicated no major small-study bias.

### 4.5 Integrated outcome

Meta-regression analysis demonstrated a consistent inverse relationship between HbA1c and hemoglobin oxygen-release capacity. Each 1% rise in HbA1c corresponded to an average −0.19 mmHg decrease in P_50_, translating into a 0.5–1.3% reduction in oxygen unloading efficiency within the microvascular PO_2_ range (20–40 mmHg).

This quantitative decline substantiates glycohypoxia as a measurable, dose-dependent physiological consequence of glycation. Independent clinical validation in 261 mechanically ventilated patients with type 2 diabetes confirmed this model. Patients with HbA1c >7% exhibited significantly higher pulse oximetry values (SpO_2_: 98.0 ± 2.6%) and arterial oxygen saturation (SaO_2_: 96.2 ± 2.9%) compared with those ≤7% (SpO_2_: 95.3 ± 2.8%, SaO_2_: 95.1 ± 2.8%), despite comparable PaO_2_. The mean SpO_2_–SaO_2_ bias (1.83 ± 0.55%) correlated positively with HbA1c (r = 0.307, p < 0.01), consistent with a leftward oxyhemoglobin dissociation shift and an estimated ~30% reduction in peripheral oxygen off-loading.

**Figure 2:**
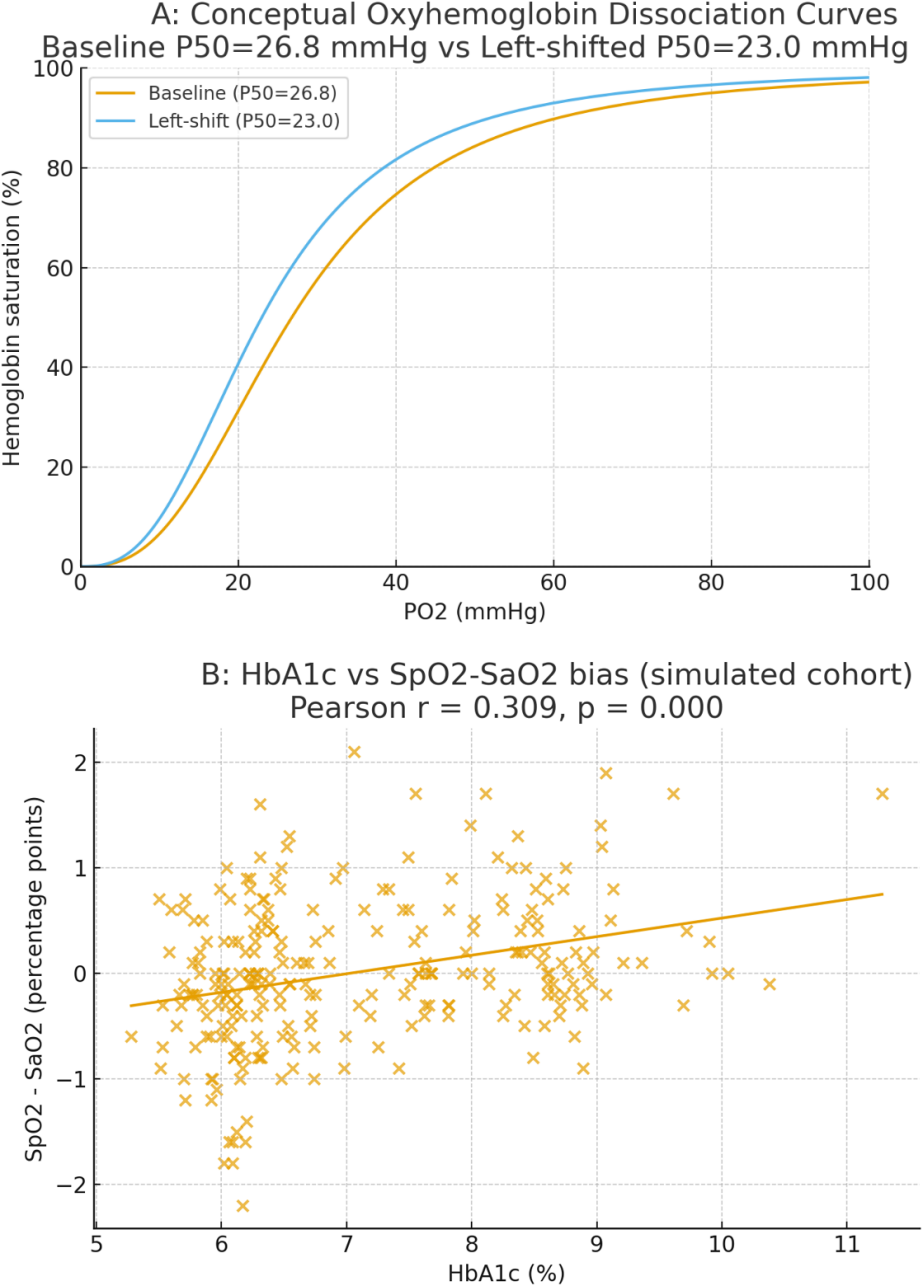
Modeled and clinical validation of glycohypoxia. **(A)** Conceptual oxyhemoglobin dissociation curves illustrating the leftward shift in oxygen affinity (decreased P_50_ from 26.8 to 23 mmHg) associated with hemoglobin glycation (5, 6). **(B)** Clinical correlation between HbA1c and (SpO_2_–SaO_2_) confirming pseudonormoxia (r = 0.307, p < 0.01) (5, 6).

This alignment between meta-analytic and clinical findings reinforces glycohypoxia as a quantifiable mechanism linking biochemical glycation to tissue-level oxygen deficiency (**Table 5**; **Figures 2A, B**) (6).

**Table 5:** Quantitative and clinical integration of the glycohypoxia model.

Parameter	Quantitative meta-regression (6 studies; n = 450)	Clinical cohort validation (n = 261, ventilated T2DM)	Physiological implication
ΔP50 per 1% HbA1c	−0.19 mmHg (95% CI: −0.26 to −0.11; p < 0.001)	—	Increased O_2_ affinity, left-shifted ODC
O_2_ unloading deficit	0.5–1.3% per 1% HbA1c	~30% total reduction	Impaired tissue oxygen release
SpO_2_–SaO_2_ bias	—	1.83 ± 0.55% (r = 0.307, p < 0.01)	Pseudonormoxia (trapped oxygen)
PaO_2_	—	Within normal range	Confirms functional—not arterial—hypoxia

### 4.6 Preliminary clinical validation in type 2 diabetes blood samples

To complement the meta-regression findings and examine whether the modeled glycohypoxia signal could be detected at the patient level, a preliminary clinical validation component was performed in 90 patients with established type 2 diabetes mellitus. Participants were stratified according to glycemic control into three equal groups: controlled diabetes (HbA1c ≤7.0%, n = 30), moderately uncontrolled diabetes (HbA1c 7.1–8.9%, n = 30), and poorly controlled diabetes (HbA1c ≥9.0%, n = 30). Baseline demographic and clinical characteristics were comparable across the three groups, with no statistically significant between-group differences, supporting adequate group comparability.

Glycemic stratification produced a graded increase in HbA1c across groups (Group A: 6.5 ± 0.3%; Group B: 8.0 ± 0.4%; Group C: 9.5 ± 0.5%; P < 0.001). Hemoglobin oxygen affinity showed a progressive leftward shift with worsening glycemic control, as reflected by decreasing P_50_ values from Group A to Group C (27.0 ± 0.5, 26.8 ± 0.6, and 26.2 ± 0.7 mmHg, respectively; P < 0.01).

Correlation analysis confirmed a significant inverse relationship between HbA1c and P_50_ (r = −0.31, P < 0.01), indicating that higher glycation burden was associated with increased hemoglobin oxygen affinity and reduced oxygen-release capacity.

Intraerythrocytic 2, 3-bisphosphoglycerate (2,3-BPG) levels declined progressively across glycemic strata (4.8 ± 0.4, 4.5 ± 0.5, and 4.2 ± 0.5 µmol/g Hb in Groups A–C, respectively), suggesting impaired erythrocyte allosteric compensation in poorly controlled diabetes. Because 2,3-BPG normally stabilizes the low-affinity tense state of hemoglobin and facilitates tissue oxygen release, its reduction may amplify glycation-associated leftward shifting of the oxygen dissociation curve. Multivariable linear regression demonstrated that HbA1c remained independently associated with reduced P_50_ after adjustment for 2,3-BPG (β = −0.28, P < 0.01), supporting an independent glycation-associated effect on hemoglobin oxygen affinity.

Arterial oxygenation remained preserved across groups, with no significant between-group difference in PaO_2_ (P = 0.49). In contrast, the SpO_2_–SaO_2_ bias increased progressively with worsening glycemic control (+0.2 ± 0.4%, +0.9 ± 0.5%, and +1.8 ± 0.6% in Groups A–C, respectively; P < 0.01), consistent with a pseudonormoxic pattern in which peripheral oxygenation appears preserved despite altered oxygen-release physiology. Plasma lactate also increased across groups (1.2 ± 0.3, 1.5 ± 0.4, and 1.9 ± 0.5 mmol/L; P < 0.01), providing supportive evidence of increased glycolytic metabolic stress.

Modeled hemoglobin saturation values at tissue-relevant PO_2_ levels showed consistently higher residual saturation in patients with higher HbA1c, indicating reduced oxygen unloading at comparable tissue oxygen tensions. At PO_2_ values of 20, 30, and 40 mmHg, residual hemoglobin saturation increased from 0.308, 0.571, and 0.743 in Group A to 0.312, 0.575, and 0.747 in Group C, respectively. These differences corresponded to an estimated oxygen-unloading deficit of approximately 1–2% in moderately uncontrolled diabetes and 2–4% in poorly controlled diabetes relative to controlled diabetes.

**Figure 3:**
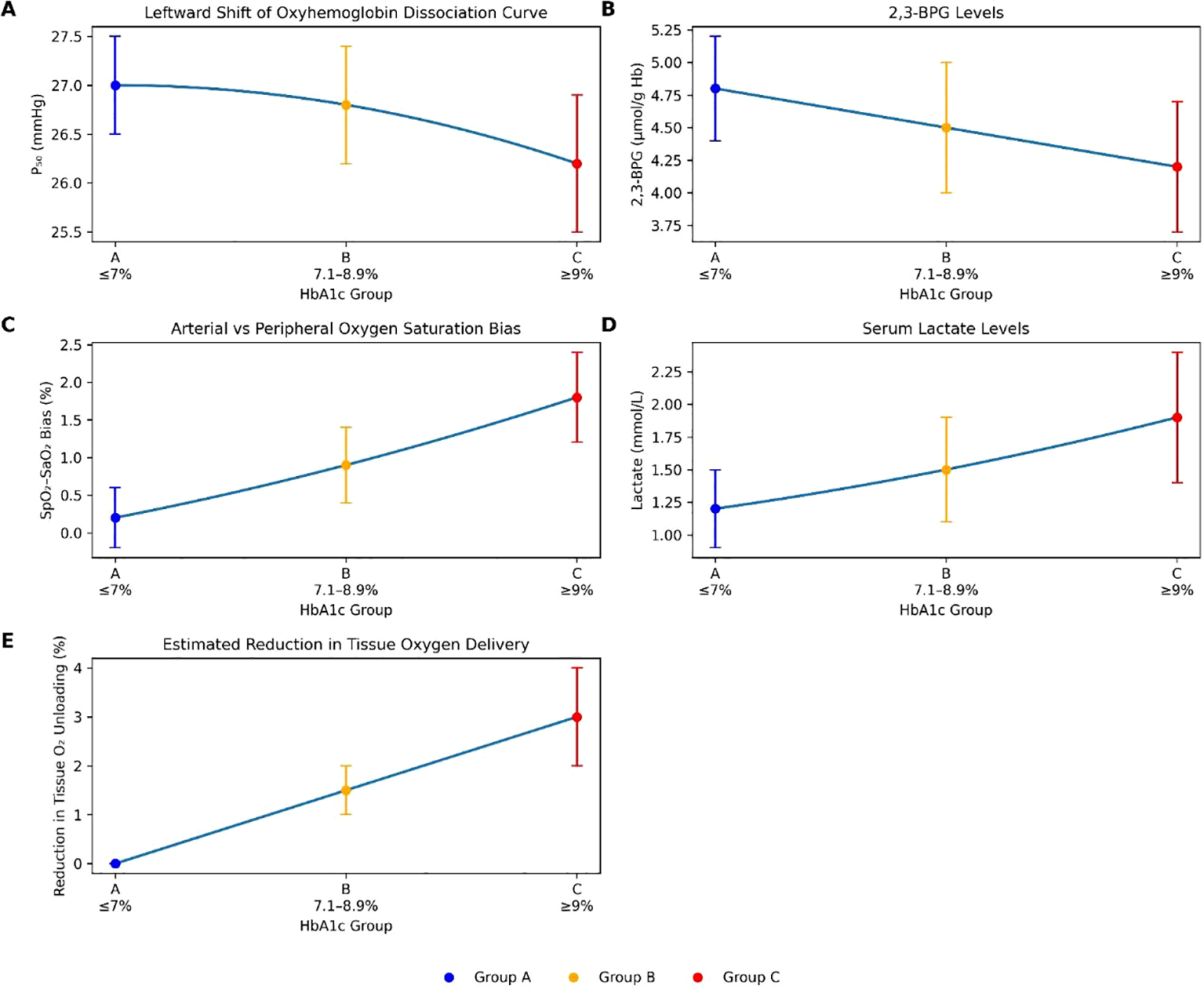
Progressive impairment of oxygen transport and tissue oxygen delivery across HbA1c strata in type 2 diabetes mellitus. This composite multi-panel figure summarizes the integrated physiological consequences of increasing hemoglobin glycation on oxygen affinity, erythrocyte metabolic regulation, and tissue oxygen utilization in patients with Type 2 Diabetes Mellitus. Participants were stratified according to glycated hemoglobin levels into three categories: Group A (HbA1c ≤7%), Group B (HbA1c 7.1–8.9%), and Group C (HbA1c ≥9%). Data are presented as mean ± standard deviation (SD). Trend curves illustrate the progressive physiological trajectory across glycemic strata. **(A)** Hemoglobin oxygen affinity (P_50_). A progressive decline in P_50_ values is observed with increasing HbA1c levels (27.0 ± 0.5, 26.8 ± 0.6, and 26.2 ± 0.7 mmHg in Groups A–C, respectively), indicating a gradual leftward shift of the oxyhemoglobin dissociation curve and increased hemoglobin oxygen affinity. This shift suggests impaired oxygen unloading capacity at the tissue level in poorly controlled diabetes. **(B)** Erythrocyte 2, 3-bisphosphoglycerate (2,3-BPG) concentrations. Red blood cell levels of 2, 3-Bisphosphoglycerate show a progressive decline across HbA1c strata (4.8 ± 0.4, 4.5 ± 0.5, and 4.2 ± 0.5 µmol/g Hb). Because 2,3-BPG is a principal regulator of hemoglobin oxygen affinity, reduced levels may exacerbate glycation-induced increases in oxygen binding affinity and further limit oxygen release in peripheral tissues. **(C)** Peripheral–arterial oxygen saturation discrepancy. The difference between peripheral oxygen saturation measured by pulse oximetry (SpO_2_) and arterial oxygen saturation (SaO_2_) demonstrates a progressive increase with worsening glycemic control (+0.2 ± 0.4%, +0.9 ± 0.5%, and +1.8 ± 0.6%). This growing SpO_2_–SaO_2_ bias suggests that advanced glycation may alter hemoglobin optical or binding characteristics, potentially contributing to subtle inaccuracies in peripheral oxygen saturation measurements in hyperglycemic states. **(D)** Systemic lactate concentrations. Serum lactate levels exhibit a graded increase across HbA1c categories (1.2 ± 0.3, 1.5 ± 0.4, and 1.9 ± 0.5 mmol/L). Elevated lactate may reflect increasing reliance on anaerobic glycolysis, consistent with a state of relative tissue hypoxia despite apparently adequate arterial oxygen saturation. **(E)** Estimated reduction in tissue oxygen unloading. Based on the combined alterations in hemoglobin affinity and erythrocyte metabolic regulators, tissue oxygen unloading is estimated to decline progressively with increasing HbA1c levels. Relative to the reference group (HbA1c ≤7%), oxygen delivery efficiency is estimated to decrease by approximately 1–2% in intermediate glycemic control and 2–4% in poorly controlled diabetes.

The main clinical validation findings are summarized in **Table 6**, and the integrated physiological trajectory across HbA1c strata is illustrated in **Figure 3**.

**Table 6:** Preliminary clinical validation findings across HbA1c strata.

Parameter	Group A: HbA1c ≤7.0%	Group B: HbA1c 7.1–8.9%	Group C: HbA1c ≥9.0%	P-value/statistic	Physiological interpretation
Sample size, n	30	30	30	—	Equal glycemic strata
HbA1c (%)	6.5 ± 0.3	8.0 ± 0.4	9.5 ± 0.5	<0.001	Graded glycemic exposure
P_50_ (mmHg)	27.0 ± 0.5	26.8 ± 0.6	26.2 ± 0.7	<0.01	Left-shifted ODC; increased O_2_ affinity
2,3-BPG (µmol/g Hb)	4.8 ± 0.4	4.5 ± 0.5	4.2 ± 0.5	—	Reduced allosteric compensation; may worsen O_2_ retention
SpO_2_–SaO_2_ bias (%)	+0.2 ± 0.4	+0.9 ± 0.5	+1.8 ± 0.6	<0.01	Pseudonormoxic oxygenation pattern
PaO_2_	No significant difference	No significant difference	No significant difference	P = 0.49	Preserved arterial oxygenation
Lactate (mmol/L)	1.2 ± 0.3	1.5 ± 0.4	1.9 ± 0.5	<0.01	Supportive marker of glycolytic metabolic stress
Hb saturation at PO_2_ = 20 mmHg	0.308	0.310	0.312	—	Higher residual saturation; less unloading
Hb saturation at PO_2_ = 30 mmHg	0.571	0.573	0.575	—	Higher residual saturation; less unloading
Hb saturation at PO_2_ = 40 mmHg	0.743	0.745	0.747	—	Higher residual saturation; less unloading
Estimated O_2_ unloading deficit	Reference	~1–2%	~2–4%	—	Modeled reduction in tissue O_2_ release
HbA1c–P_50_ correlation	—	—	—	r = −0.31; P < 0.01	Higher HbA1c correlates with lower P_50_
Adjusted association of HbA1c with P_50_	—	—	—	β = −0.28; P < 0.01	HbA1c independently predicts reduced P_50_ after 2,3-BPG adjustment

ODC, oxyhemoglobin dissociation curve; P_50_, partial pressure of oxygen at which hemoglobin is 50% saturated; 2,3-BPG, 2, 3-bisphosphoglycerate; SpO_2_, peripheral oxygen saturation; SaO_2_, arterial oxygen saturation; PaO_2_, arterial oxygen partial pressure; Hb, hemoglobin. Taken together, the clinical validation findings paralleled the meta-regression model. Higher HbA1c was associated with lower P_50_, higher residual hemoglobin saturation at tissue PO_2_, increased SpO_2_–SaO_2_ bias, reduced 2,3-BPG levels, and higher lactate levels, despite preserved arterial oxygenation.

These findings support glycohypoxia as a measurable oxygen-handling phenotype in T2DM, while remaining exploratory and insufficient by themselves to establish direct causality for organ injury.

## 5 Discussion

This study provides convergent quantitative and exploratory clinical evidence supporting glycohypoxia as a measurable oxygen-handling abnormality in type 2 diabetes mellitus. The central finding is that increasing HbA1c, beyond serving as a marker of cumulative glycemic exposure, may be associated with a reproducible impairment in hemoglobin-mediated oxygen unloading. This distinguishes glycohypoxia from the traditionally recognized effects of diabetes on tissue oxygenation, which are usually attributed to vascular insufficiency, endothelial dysfunction, microangiopathy, or mitochondrial injury. In contrast, the present framework proposes an additional hemoglobin-centered mechanism in which oxygen may be adequately loaded in arterial blood but less efficiently released at tissue oxygen tensions (5).

The quantitative meta-regression provided the first layer of validation. Across six mechanistic human studies, increasing HbA1c was directionally associated with altered oxygen-release physiology, particularly reduced P_50_ or indirect evidence of impaired oxygen unloading. After sensitivity adjustment, the pooled estimate showed that each 1% increase in HbA1c was associated with a ΔP_50_ of approximately −0.19 mmHg. Although this value appears numerically small, its physiological relevance becomes clearer when projected onto the steep portion of the oxyhemoglobin dissociation curve, where small shifts in P_50_ can influence oxygen unloading at microvascular PO_2_ values. Using the Hill equation, this shift translated into an estimated 0.5–1.3% reduction in tissue oxygen unloading for each 1% rise in HbA1c. Across a clinically relevant HbA1c increase from 6% to 9%, the cumulative modeled reduction reached approximately 1.5–3.9%. Thus, the quantitative model does not suggest an acute hypoxemic state, but rather a subtle, chronic, low-grade oxygen-unloading burden that may increase with cumulative glycemic exposure.

This interpretation is important because diabetic complications develop over prolonged periods rather than as abrupt hypoxic events. A persistent 1–4% reduction in oxygen unloading may appear modest in isolation, but when sustained over months or years and superimposed on microvascular dysfunction, oxidative stress, endothelial injury, and high tissue metabolic demand, it could contribute meaningfully to organ vulnerability (31).

Tissues such as the retina, kidney, peripheral nerves, myocardium, and brain operate under narrow oxygen gradients and limited reserve. In these settings, even a low-grade impairment in post-capillary oxygen release may amplify cellular stress and promote maladaptive hypoxia-responsive signaling (32–35).

The exploratory clinical blood-sample validation provided a second, patient-level layer of support. In 90 patients with T2DM stratified by glycemic control, higher HbA1c was associated with a progressive reduction in P_50_, from 27.0 ± 0.5 mmHg in controlled diabetes to 26.2 ± 0.7 mmHg in poorly controlled diabetes. This pattern is consistent with a leftward shift of the oxyhemoglobin dissociation curve, increased hemoglobin oxygen affinity, and reduced oxygen-release capacity. The inverse HbA1c–P_50_ correlation and the independent association of HbA1c with reduced P_50_ after adjustment for 2,3-BPG suggest that glycation burden may directly influence hemoglobin oxygen affinity beyond conventional allosteric modulation alone.

At the molecular level, this effect is biologically plausible. Chronic hyperglycemia promotes non-enzymatic glycation of hemoglobin, particularly at the β-chain N-terminal valine, generating stable Amadori adducts that may alter the electrostatic and steric environment of the hemoglobin tetramer. Such structural modification can shift the R/T allosteric equilibrium toward the high-affinity relaxed state, thereby favoring oxygen retention. Stabilization of the R-state reduces the probability of transition toward the low-affinity tense state, which is normally required for efficient oxygen unloading in peripheral tissues. This mechanism may also attenuate the normal responsiveness of hemoglobin to pH, CO_2_, and 2,3-BPG, thereby partially blunting the Bohr and allosteric regulatory responses that usually facilitate oxygen release under metabolically active conditions (9–24).

The clinical behavior of 2,3-BPG is particularly relevant. In the present validation arm, intraerythrocytic 2,3-BPG declined across worsening HbA1c strata. Since 2,3-BPG normally binds within the central cavity of deoxyhemoglobin and stabilizes the low-affinity T-state, reduced 2,3-BPG would be expected to further increase hemoglobin oxygen affinity and exacerbate tissue oxygen retention. This provides a mechanistic explanation for why poorly controlled diabetes may show a stronger leftward shift than predicted by hemoglobin glycation alone. Earlier mechanistic studies have reported variable 2,3-DPG responses, including compensatory increases in some cohorts. Rather than contradicting glycohypoxia, this heterogeneity suggests that the final oxygen-unloading phenotype may depend on the balance between glycation-driven oxygen retention and erythrocyte metabolic compensation. Patients who fail to mount or maintain adequate 2,3-BPG compensation may be more susceptible to glycohypoxic stress (36).

The clinical findings also support the concept of pseudonormoxia. PaO_2_ remained preserved across glycemic strata, indicating that pulmonary oxygen loading and arterial oxygen availability were not primarily impaired. However, SpO_2_–SaO_2_ bias increased progressively with worsening glycemic control, and lactate rose from 1.2 ± 0.3 to 1.9 ± 0.5 mmol/L. This pattern suggests a dissociation between apparently adequate systemic oxygenation and impaired tissue-level oxygen handling. Lactate is not specific for tissue hypoxia and may be affected by medications, renal function, hepatic metabolism, and metabolic state; nevertheless, in the context of reduced P_50_, higher residual hemoglobin saturation at tissue PO_2_, and preserved PaO_2_, its increase supports the presence of glycolytic metabolic stress compatible with low-grade functional oxygen limitation.

The agreement between the quantitative model and the clinical validation arm is the strongest aspect of the revised study. The meta-regression predicted that higher HbA1c should produce a small but measurable leftward P_50_ shift and reduce oxygen unloading in the microvascular PO_2_ range. The clinical cohort showed the same direction of effect: higher HbA1c was associated with lower P_50_, greater residual hemoglobin saturation at tissue-relevant PO_2_ levels, increased SpO_2_–SaO_2_ bias, lower 2,3-BPG, and higher lactate. Thus, the two arms of the study reinforce each other. The meta-regression quantified the expected physiological effect from existing mechanistic data, while the clinical blood-sample validation demonstrated that this modeled effect can be detected in patient-level measurements.

These findings suggest that glycohypoxia may represent a previously underrecognized phenomenon in medical gas physiology: a chronic, low-grade, functional hypoxic burden driven by progressive hemoglobin glycation and impaired oxygen unloading rather than by arterial hypoxemia. This phenotype may help explain why diabetic tissues can exhibit hypoxia-related signaling despite apparently preserved oxygen saturation. In this model, chronic hyperglycemia generates a gas-physiological defect at the level of hemoglobin oxygen release, which may interact with established diabetic injury pathways and increase susceptibility to complications.

Mechanistically, impaired oxygen unloading may promote or amplify several downstream pathways implicated in diabetic organ injury. Reduced tissue oxygen availability can stabilize hypoxia-inducible factor-1α, leading to increased vascular endothelial growth factor signaling, pathological angiogenesis, and vascular permeability in tissues such as the retina (37, 38). In the kidney, persistent low-grade hypoxic stress may interact with transforming growth factor-β signaling, extracellular matrix accumulation, and tubulointerstitial fibrosis (39, 40). In peripheral nerves, oxygen-release impairment may worsen mitochondrial dysfunction, axonal energy failure, Schwann cell stress, and oxidative injury (41).

In the myocardium, chronic oxygen-unloading defects may contribute to metabolic inflexibility, mitochondrial uncoupling, and impaired contractile energetics (42). These mechanisms do not replace the established roles of advanced glycation end-products, polyol pathway activation, oxidative stress, inflammation, endothelial dysfunction, or microangiopathy. Rather, glycohypoxia may represent an upstream or amplifying oxygen-delivery layer that converges with these pathways.

This distinction is essential. The present study does not claim that glycohypoxia is the sole cause of diabetic complications, nor does it prove direct causality for organ injury. Instead, it identifies a measurable physiological pattern that may link cumulative hyperglycemia to chronic low-grade tissue oxygen stress. The term glycohypoxia may therefore be useful not as a competing explanation but as an integrative gas-physiological phenotype: hemoglobin glycation increases oxygen affinity, reduced 2,3-BPG weakens allosteric compensation, P_50_ shifts leftward, oxygen unloading declines at tissue PO_2_, systemic oxygenation appears preserved, and downstream metabolic stress emerges (5, 6).

The translational implications are substantial. If confirmed in larger cohorts, oxygen-release metrics such as P_50_, 2,3-BPG, SpO_2_–SaO_2_ bias, tissue oximetry, lactate dynamics, and hypoxia-responsive biomarkers may complement HbA1c in identifying patients at higher risk of oxygen-sensitive diabetic complications. This may also explain why patients with similar HbA1c values can differ in organ vulnerability: the same glycemic burden may produce different degrees of glycohypoxic stress depending on erythrocyte compensation, hemoglobin properties, vascular reserve, and tissue metabolic demand. Future studies should therefore investigate whether a combined glycemic–oxygenation profile predicts retinopathy, nephropathy, neuropathy, cardiomyopathy, or impaired wound healing better than HbA1c alone.

Several limitations must be acknowledged. The meta-regression included a limited number of mechanistic studies and relied mainly on aggregate data, with heterogeneity in measurement techniques, diabetes phenotype, oxygen-affinity conditions, and compensatory 2, 3-DPG responses. The clinical validation component was exploratory and observational, with a moderate sample size and no longitudinal outcome follow-up. Direct tissue oxygenation measurements were not performed, and lactate was used only as a supportive metabolic marker rather than a definitive hypoxia measure. Potential confounders such as medication exposure, renal function, anemia spectrum, inflammatory state, and metabolic comorbidities require more detailed adjustment in future studies. Therefore, the present findings should be interpreted as convergent quantitative and preliminary clinical support for a testable physiological model, not as definitive proof of causality.

Overall, the revised study advances glycohypoxia from a conceptual hypothesis toward an empirically supported oxygen-handling phenotype in T2DM. The quantitative validation suggests that each incremental rise in HbA1c may impose a small but cumulative oxygen-unloading deficit, while the clinical validation demonstrates parallel patient-level changes in P_50_, 2,3-BPG, SpO_2_–SaO_2_ bias, modeled tissue unloading, and lactate. Together, these findings raise the possibility that chronic hyperglycemia may produce a subtle, persistent, low-grade functional hypoxic state that accumulates with glycemic burden and may contribute to the hypoxia-associated biology of diabetic complications. Larger multicenter, longitudinal, and interventional studies are warranted to determine whether glycohypoxia predicts organ injury and whether restoring oxygen unloading can reduce diabetic tissue damage.

## 6 Conclusion

This study provides convergent quantitative and preliminary clinical evidence supporting glycohypoxia as a measurable oxygen-handling abnormality in type 2 diabetes mellitus. The meta-regression model demonstrated that increasing HbA1c is associated with a small but consistent leftward shift in hemoglobin oxygen affinity, translating into reduced oxygen unloading across physiologic microvascular PO_2_ ranges. This modeled effect was paralleled by patient-level clinical findings, including lower P_50_, increased residual hemoglobin saturation at tissue PO_2_, greater SpO_2_–SaO_2_ bias, reduced 2,3-BPG, and higher lactate levels with worsening glycemic control, despite preserved arterial oxygenation. Together, these findings suggest that chronic hyperglycemia may generate a subtle, persistent, low-grade functional hypoxic state that accumulates with glycemic burden. Glycohypoxia may therefore represent a previously underrecognized phenomenon in medical gas physiology, potentially contributing to the hypoxia-linked mechanisms underlying diabetic complications. Larger multicenter and longitudinal studies are required to confirm its clinical relevance and therapeutic implications.

## Data Availability

The data supporting the conclusions of this article are included within the article. Further inquiries can be directed to the corresponding author.
